# Sex Differences in Bench Press Strength and Power: A Velocity-Based Analysis Adjusted for Body Composition

**DOI:** 10.3390/jfmk10030284

**Published:** 2025-07-24

**Authors:** Olga López-Torres, Raúl Nieto-Acevedo, Amelia Guadalupe-Grau, Valentín Emilio Fernández Elías

**Affiliations:** 1 Department of Sports Sciences, Faculty of Medicine, Health and Sports, Universidad Europea de Madrid, 28679 Villaviciosa de Odón, Spain; olga.lopez@universidadeuropea.es; 2 Faculty of Sports Sciences, Universidad Alfonso X el Sabio, 28691 Villanueva de la Cañada, Spain; racevnie@uax.es; 3 GENUD Toledo Research Group, Faculty of Sports Sciences, Universidad de Castilla–La Mancha, Avda, Carlos III S/N, 45071 Toledo, Spain; 4 CIBER on Frailty and Healthy Aging (CIBERFES), Instituto de Salud Carlos III, 28029 Madrid, Spain; 5 Research Centre in Sport Sciences, Rey Juan Carlos University, 28943 Fuenlabrada, Spain; valentin.fernandez@urjc.es

**Keywords:** sex characteristics, muscle strength, resistance training, fat-free mass, power output, velocity-based training

## Abstract

**Background:** Resistance training (RT) promotes muscle hypertrophy and strength gains in both men and women. However, sex differences in neuromuscular performance, muscle fiber composition, and the hormonal environment influence strength and power adaptations. While men generally exhibit greater absolute and relative strength, it remains unclear to what extent these differences persist across various load intensities. A better understanding of sex-specific strength and power profiles may help optimize training strategies. The aim of this study was to compare strength and power performance during the bench press exercise in physically active males and females, relative to body mass and fat-free mass (FFM). **Methods:** Twenty-nine physically active individuals (16 men: 21.3 ± 4.1 years, 13 women: 22.6 ± 4.9 years) performed a one-repetition maximum (1RM) test and an incremental velocity-based assessment at 45%, 55%, 65%, 75%, and 85% of the 1RM using a Smith machine. The barbell velocity was measured via a linear transducer, with the mean propulsive velocity (MPV) recorded for each load. Power-related variables (e.g., peak force [F0], maximal velocity [V0], and maximal power [Pmax]) were analyzed. To account for differences in body composition, data were adjusted for body mass and FFM. **Results:** Men exhibited significantly greater strength and power than women across most loads when adjusted for both body mass and fat-free mass (FFM) (*p* < 0.05). These differences were particularly pronounced when normalized to FFM (45–75%1RM; *p* = 0.001–0.031), with large effect sizes observed (ηp^2^ = 0.185–0.383). Notably, sex differences in mean propulsive velocity (MPV) disappeared at 85%1RM (*p* = 0.208; ηp^2^ = 0.06), suggesting that maximal neuromuscular recruitment may minimize sex-related disparities at higher intensities. Furthermore, men demonstrated significantly higher values in six of the seven power-related variables, with no significant differences in the %1RM required to achieve an optimal power output. **Conclusions:** These findings confirm that men exhibit greater strength and power than women, even after adjusting for body composition. However, at high relative loads (≥85%1RM), sex differences in movement velocity appear to diminish, likely due to similar recruitment patterns of high-threshold motor units. These results highlight the importance of sex-specific resistance training programs, particularly in relation to load prescription and the application of velocity-based training methods.

## 1. Introduction

Long-term resistance training (RT) is known to induce muscle hypertrophy and strength gains in both men and women, although responses can vary widely between individuals [1,2]. Historically, RT research has focused predominantly on male populations, but recent efforts have expanded to include healthy adult women outside of clinical contexts such as menopause, osteoporosis, or fibromyalgia [3].

Biological differences between sexes—such as hormonal profiles, muscle fiber composition, and substrate utilization—can influence adaptations to resistance training [4]. For example, women generally have lower testosterone levels, greater insulin sensitivity, and different muscle fiber distribution, which may affect strength and hypertrophy outcomes [5]. Furthermore, fluctuations in estrogen levels across the menstrual cycle may modulate neuromuscular performance, with evidence suggesting enhanced strength gains during the follicular phase [6]. While some studies have suggested that fluctuations in estrogen and progesterone across the menstrual cycle may influence neuromuscular performance, recent meta-analyses and large-scale reviews indicate that these effects are generally small, inconsistent, and highly individualized [7,8]. However, hormonal contraceptive use may alter or blunt these fluctuations, potentially leading to more stable performance profiles [9]. Therefore, although the menstrual cycle phase is not universally considered a major confounder in resistance training studies, accounting for it may still be relevant in protocols where hormonal status could modulate outcomes, particularly when investigating sex-based performance differences.

Traditionally, training loads have been prescribed using the one-repetition maximum (1RM), a method that presents notable limitations such as inter-day variability and limited sensitivity to fatigue-induced performance changes [10,11]. In contrast, Velocity-Based Training (VBT) has gained prominence as a more dynamic and individualized approach. By monitoring the velocity of the concentric phase of each repetition, VBT allows for the real-time adjustment of training intensity based on the athlete’s neuromuscular status [12]. This method has been shown to enhance strength and power adaptations, reduce overtraining risk, and improve training specificity, particularly in high-performance settings [13,14,15]. Additionally, VBT has proven effective in identifying individual strength–velocity profiles and load–velocity relationships, which are essential for personalized programming [16,17]. Despite growing interest in VBT, few studies have systematically examined the sex differences in velocity-based strength and power performance using standardized protocols.

It is well established that men typically exhibit higher absolute and relative strength than women [14]. However, the extent to which these differences persist when performance is normalized to body mass or fat-free mass (FFM)—particularly across a range of submaximal loads—remains unclear. Given that power (the product of force and velocity) is a critical component in both athletic performance and functional capacity, understanding sex-specific strength–velocity profiles is essential [15].

Few studies have standardized velocity-based training (VBT) protocols to comprehensively explore sex differences [16]. Biological variability within subjects can be significantly reduced by implementing a controlled pause between the eccentric and concentric phases of movement [17,18,19]. Therefore, this study aims to examine the sex-based differences in maximal concentric velocity during the bench press exercise using a standardized protocol that includes a controlled pause and accounts for the menstrual cycle phase [20,21]. The bench press was chosen due to its widespread application in strength training and sport performance testing, particularly for assessing upper-body strength, where sex differences are more pronounced [22]. Accordingly, the aim of this study was to compare the strength and power performance between male and female athletes, relative to body mass and fat-free mass. We hypothesized that males would demonstrate higher maximum strength and power values relative to body mass and fat-free mass compared to females.

## 2. Materials and Methods

### 2.1. Experimental Approach to the Problem

The present study was a cross-sectional, not controlled, not blinded, non-randomized study, designed to analyze the sex differences in the bench press exercise performed at maximal concentric velocity. Two different testing sessions were carried out, separated by 48–72 h by the same evaluators between January and March 2021 in the muscle performance laboratory of the Faculty of Sport Sciences, University of Castilla-La Mancha, Toledo, Spain. In the first session, participants performed a 1RM bench press exercise test using a Smith machine (a barbell that is fixed within steel rails allowing only vertical or near-vertical movement within a steel frame). In the second session, volunteers performed an incremental test against five different loads (45%, 55%, 65%, 75%, and 85% of the 1RM) at maximal concentric velocity to analyze the load-velocity profile. Subjects performed three repetitions against each load and the mean propulsive velocity (MPV) of the barbell was measured with a linear velocity transducer (T-Force system, Ergotech), considered as the gold standard. Briefly, MPV represents the value from the start of the concentric phase to the moment the acceleration of the bar is lower than gravity (−9.81 m·s^−2^) [23]. The protocol performed by males and females was identical.

### 2.2. Subjects

A priori sample-size estimation was conducted using G*Power 3.1 for an ANCOVA design with two groups and two covariates (body mass and FFM). Based on the effect-size estimates for the sex differences in the mean propulsive velocity from previous studies (ηp^2^ ≈ 0.14, large), and aiming for 80% power at α = 0.05, the analysis indicated a minimum of 26 participants. Allowing for potential dropouts, we enrolled 29 subjects (15 men and 14 women). Therefore, twenty-nine participants (*n* = 16 men, mean age ± SD 21.3 ± 4.1 years and *n* = 13 women, age ± SD 22.6 ± 4.9 years) volunteered to join the study. Participants with previous resistance exercise experience were recruited from the university student population, (2.66 ± 1.83 years), and all of them were familiarized with the bench press exercise technique before the beginning of the study. The volunteers presented no health problems, no musculoskeletal injuries, and did not suffer from physical limitations that could compromise the study. The subjects were asked to avoid any strenuous exercise 48 h before each testing session. They were informed of the study procedures and signed a written informed consent form before initiating the study. No dropouts or missing data were reported. The study protocol adhered to the tenets of the Declaration of Helsinki and was approved by the Ethics Committee of the Universidad de Castilla-La Mancha (UCLM) with the approval code CEIC924 in January 2021.

### 2.3. Procedures

Body composition information was collected on the first day of testing using bioimpedance (BIA) measures (Tanita BC 418-MA, Tanita Corp., Tokyo, Japan), followed by a standardized warm-up consisting of 10 min of aerobic exercise (jogging, rowing, or cycling by choice), three sets of isometric upper-body exercises, and three sets of 10 repetitions of the bench press exercise with a 20 kg bar. The participants were encouraged to perform the last three repetitions of each set of 10 at maximal concentric velocity. After warming up, the participants performed an incremental loading bench press maximum strength test (1RM) in a Smith machine. To execute the bench press, the volunteers were laying on the bench placed under the rack where the bar rested. They could decide the preferred distance to grab the bar while maintaining bent elbows. Keeping the scapula on the bench, the participants were encouraged to fully extend their elbows when raising the bar, imposing a pause between the concentric and the eccentric phases. The load added to the bar was increased by 10 kg when the velocity was over 0.50 m/s, 5 kg when the velocity was between 0.49 and 0.25 m/s, and 2 kg when the velocity was under 0.25 m/s [20]. For each load, participants performed two repetitions except for the 1RM set where they could only perform one. Between sets, the subjects rested for 3 min. To avoid circadian variations, all sessions were carried out in the morning. In addition, to standardize the hormone situation, women performed the first session on the first day of their menstrual bleeding phase. Therefore, all female participants performed the two study sessions during their menstrual bleeding phase (the first and the second–third day). Although there is some evidence that sport performance can be reduced during the bleeding phase of the menstrual cycle [24,25,26], due to testing limitations and inconclusive results from the literature [27,28], the easiest and most precise way to standardize the menstrual phase for all female participants was to use the bleeding phase.

On the second day of testing and after warming up similarly to session one, participants performed the bench press exercise in the Smith machine against five loads (45%, 55%, 65%, 75%, and 85% of the 1RM load obtained in the first session) in an incremental order. The subjects imposed a pause between the concentric and the eccentric phases to reduce the biological within-subject variability [29]. After the eccentric phase, participants released the barbell weight on the safety stops for two seconds (controlled with a stopwatch) while still grabbing the barbell. Thereafter, the researcher gave the subject an acoustic signal to start a purely concentric push at the maximum possible velocity. In between sets, participants rested for three minutes, individually choosing between laying or sitting on the bench or standing/walking. The participants performed three repetitions with each load.

### 2.4. Measurement Equipment and Data Acquisition

The technical characteristics of the device used for measuring the barbell mean propulsive velocity (MPV) of each repetition performed are shown in Table 1. The device operates as a linear velocity transducer (no mobile app support) and calculates output metrics indirectly from velocity and time measurements (including the peak force, mean velocity, mean power, time to peak power, propulsive phase duration, estimated %1RM, predicted 1RM, number of repetitions, velocity loss (%), and velocity-based alerts). A low repetition-loss rate of 0.8 per 100 trials underscores its reliability. For the study, the T-Force device was placed vertically under the barbell and connected to a laptop to immediately obtain the data. In addition, the reproducibility of the same testing protocol with the T-Force system had been evaluated in a previous study conducted by our group [30], showing a high test–retest reliability across key performance variables. These results were consistent with those reported by other authors using the same device and methodology in resistance-trained populations [31].

### 2.5. Variables Analyzed

The dependent variables analyzed included the mean propulsive velocity (MPV) of the bar measured at the five different %1RM (45%, 55%, 65%, 75%, and 85%), as well as seven different variables obtained from the excel sheet proposed by Alcazar et al. [32]. This excel sheet registers the MPV measured by an external device (T-force linear encoder, in this concrete study) to posteriorly calculate the following: initial force (F0), which measures, in Newtons, the maximal applied to the bar before it starts moving during isometric contraction; final velocity (V0), which measures, in m/s, the maximal bar displacement before stopping at the end of the concentric phase; Maximal power (Pmax), which measures, in watts, the maximal power achieved; Optimal force (Fopt), which measures, in Newtons, the optimal force that must be applied to specifically train power; Optimal velocity (Vopt), which measures, in m/s, the optimal velocity to be moving the bar to specifically train power; %1RM at Pmax, which measures which % of 1RM should be applied, in kg, to specifically train power; and Optimal load (Lopt), which measures the optimal load, in kg, that should be lifted to specifically train power.

### 2.6. Statistical Analysis

The data normality was assessed using the Shapiro–Wilk test prior to the inferential analyses, confirming that all variables were normally distributed; then, parametric analyses were considered for subsequent comparisons to assess the differences between sexes. Secondly, in order to compare men and women participants, an ANCOVA model was run, including all the dependent variables (measures) as well as the 1RM (kg) and Fat-Free Mass (FFM) (kg) as covariates. Due to the identification of a statistically significant positive correlation between both sexes (r = 0.576; *p* = 0.019 and r = 0.640; *p* = 0.018 for men and women, respectively), two independent ANCOVA models were used for the 1RM (kg) and FFM (kg), respectively, in order to avoid collinearity problems. The effect sizes for ANCOVA were reported as partial eta squared (ηp^2^) and were interpreted according to Cohen’s benchmarks: small (ηp^2^ ≥ 0.01), medium (ηp^2^ ≥ 0.06), and large (ηp^2^ ≥ 0.14). When paired comparisons were performed, Cohen’s d was calculated and classified as trivial (<0.20), small (0.20–0.49), moderate (0.50–0.79), and large (≥0.80). All the analyses were run using the statistical software IBM SPSS for Windows, version 26.0 (Armonk, NY, USA: IBM. Corp.), and the significance level was set at *p* < 0.05.

## 3. Results

The participant’s descriptive characteristics are summarized in Table 2. The mean ± SD values for the mean propulsive velocity (MPV) at each %1RM and for all power-related variables in men and women are shown in Table 3. When the 1RM (kg) relative to total body mass was included as a covariate, significant sex differences emerged only at 65%1RM (*p* = 0.022) and 75%1RM (*p* = 0.046). In contrast, using the 1RM/FFM as the covariate revealed significant differences at 45% (*p* = 0.022), 55% (*p* = 0.031), 65% (*p* < 0.001), and 75% of the 1RM (*p* = 0.011), with large effect sizes in all cases (ηp^2^ = 0.166–0.383). No differences were observed at 85%1RM for either covariate.

For power variables (Table 4), six of the seven measures differed significantly between sexes (all *p* ≤ 0.05), except for the %1RM at Pmax. The effect sizes were large when adjusting for body mass (ηp^2^ > 0.110) and very large when adjusting for the FFM (ηp^2^ > 0.200).

## 4. Discussion

The aim of the present study was to compare the relative strength and power performances in males and females, considering both their body-weight-adjusted (1RM/kg) and fat-free mass-adjusted (1RM/FFM) ratios, along with velocity-based power outputs. The analysis conducted revealed that, for most of the variables studied, men exhibited higher relative strength values than women in both ratios examined (1RM/kg and 1RM/FFM), with the differences being more pronounced when using the 1RM/FFM ratio. Additionally, in all power-related variables analyzed, males demonstrated higher values, except for their %RM. The lack of significant differences in the %RM between sexes suggests that there is an optimal percentage of RM for power training that is consistent and independent of sex. Differences between sexes in the power accounted for muscle fiber types, muscle quality, or glycolytic enzymatic activities [33]. A qualitative difference in muscle tissue, such as a higher concentration of glycolytic enzymes and a greater proportion of fast type muscle fibers, may explain the disparity in strength [34], which might also explain why the differences were more pronounced when adjusted by FFM.

This is consistent with Bartolomei et al. [35], who detected a significantly greater 1RM in the bench press adjusted for FFM in men than in women. As suggested by previous investigations [36], FFM can be considered as one of the most important factors for maximal strength and power performance. Although, no significant sex differences after adjusting for FFM were detected for 1RM in the squat exercise [35]. Sex differences are obvious in all muscle groups but are larger for upper-body than lower-body muscle. For this reason, the difference in absolute strength between sexes appears more evident in the upper body compared to the lower body [22].

Moreover, the results of this study revealed significant differences between sexes in the MPV at most relative loads to the 1RM (45%, 55%, 65%, and 75%) as well, whereas these differences were not significant at 85%1RM. These observations can be explained by the physiological and neuromuscular factors inherent to each sex, as well as the differences in muscle fiber recruitment strategies under varying load levels. Similar observations were found by others who evaluated the bench press, military press, squat, and row [37,38,39,40]. Similarly, other meta-analyses have shown that sex differences in relative loading disappear at high intensities (>85–90%1RM) [41]. Furthermore, Izadi et al. [42] found that women exhibited reduced velocities when handling lighter relative loads compared to men. Conversely, women demonstrated higher velocities when dealing with loads exceeding 85% of their 1RM in contrast to their male counterparts. The significant differences observed at the loads from 45% to 75% of the 1RM align with previous research highlighting men’s greater capacity for force and power production due to their higher proportion of type II muscle fibers, higher testosterone levels, and greater muscle cross-sectional area [14,22]. Maximal strength and power are influenced by many neuromuscular factors including muscle morphological characteristics such as muscle thickness, the pennation angle, and fascicle length [35], as well as fiber type [22]. These characteristics enable men to generate greater explosive force and velocity during repetitions performed with low to moderate loads. Additionally, the use of the 1RM/FFM ratio as a covariate highlights that the differences are not solely attributable to total body mass but also to FFM composition, which is higher in men. On the other hand, women tend to exhibit greater fatigue resistance at lower loads due to their reduced reliance on type II fibers and greater use of type I fibers, along with a lower accumulation of metabolites during repetitive contractions [43]. However, these advantages are insufficient to match the MPV generated by men at these loads, explaining the observed differences. The absence of significant differences at 85%1RM may be attributed to the convergence in muscle recruitment strategies as the load increases. At higher loads, both men and women activate a greater percentage of their available muscle fibers, including both the fast-twitch type II and slow-twitch type I fibers [35]. This reduces the disparity between sexes, as structural and hormonal differences have a less pronounced impact at these load levels, where neuromuscular factors are primarily limiting. Additionally, recent studies have indicated that women demonstrate superior relative efficiency in handling loads close to their maximum [42]. This adaptation may be related to a greater capacity for sustained effort and reduced strength deficits during prior eccentric contractions, contributing to equalizing velocities with men at this load.

Interestingly, we did not obtain significant differences in the optimal power in bench press (57.18 ± 1.56% of the 1RM for men; 58. 61± 5.42 of the 1RM for women). These results are different from those obtained by Thomas et al., [44] who found differences between sexes in the optimal power output during the squat jump (30-40% of the 1RM for men; 30-50% of the 1RM for women) and bench throw (30% of the 1RM for men; 30-50% of the 1RM for women) exercises. However, it is worth noting that the ranges reported by Thomas et al. partially overlap (e.g., both sexes include 30%), and differences may only emerge when comparing extreme values (e.g., 30% vs. 50%). Alonso-Aubin et al. [45] also found significant sex-related differences in the bench press exercise for power and time to maximum velocity (40–60–70–80%). Moreover, Bartomei et al. [35] found that lower levels of power were detected in females in the upper body (−61.2%). These results could be explained in part by the difference between sexes in anaerobic power, regardless of their FFM. Similar results have been previously reported by Mayhew et al. [46] and Perez-Gomez et al. [47]. These results differ slightly from those reported by Torrejón et al. [48]. Although they found differences in the 1RM between men and women, these differences disappeared when the load was adjusted for body mass. Our results and the results obtain by Torrejón et al. [48] reveals that sex differences are still evident when power per kg of body mass is considered. Consequently, future research should take into account Fat-Free Mass when comparing strength and power between genders.

Equally, we demonstrated that the P_max_, F_0_, and V_0_ differed significantly between males and females. These results are in line with Nikolaidis [49], who found that boys had higher values of P_max_, rP_max_, and V_0_ than girls, while no differences were found for F_0_ and V_0_/F_0_. Thus, the physiological differences explained previously (i.e., muscle enzyme activities, electromyographic activity, muscle fiber composition, etc.) could explain these differences as well.

By integrating relative-strength and velocity-based power metrics, our study provides a coherent picture: the sex differences in neuromuscular performance are multifactorial, arising from both quantitative (FFM) and qualitative (fiber-type composition, hormone milieu) distinctions. Importantly, while normalizing to FFM attenuates, but does not abolish, sex gaps in strength, velocity-power outputs at submaximal loads remain consistently higher in men. This suggests that training prescriptions aiming to optimize power should consider sex-specific loading zones and recovery strategies, given women’s relatively greater fatigue resistance at lighter loads. Subsequent research should employ longitudinal interventions to determine whether targeted hypertrophy or neural-adaptation programs can minimize these sex disparities, particularly by enhancing type II fiber recruitment in women. Moreover, exploring the hormonal fluctuations across menstrual cycles may elucidate acute changes in force–velocity profiles. Finally, expanding the scope to include dynamic, sport-specific movements will help translate laboratory findings into practical performance gains.

## 5. Practical Implications

These findings highlight the importance of individualizing resistance training programs based on sex-specific force–velocity profiles. For instance, women may benefit from targeted work with moderate loads to enhance movement velocity and power output, while men may emphasize the development of maximal force production with higher loads, given their greater absolute strength capacity. Such distinctions can inform more effective and tailored programming strategies for performance optimization.

## 6. Limitations and Future Research

Although Fat-Free Mass was assessed using bioelectrical impedance analysis (BIA) rather than the reference method of dual-energy X-ray absorptiometry (DEXA), recent large-scale evidence from the UK Biobank indicates that BIA provides comparable estimates of body composition at the group level when standardized protocols are applied [50]. Nevertheless, we acknowledge this as a methodological limitation and recommend the use of DEXA in future studies for improved accuracy.

Also, although the reliability of the T-Force system had been established in the prior literature, we did not conduct a formal test–retest reliability assessment within the current sample. However, our study strictly adhered to standardized testing protocols, and previous research from our group using this same device and procedure demonstrated excellent consistency in repeated measurements. These findings are supported by other studies reporting the high reliability of the T-Force system for velocity-based training metrics [30,31]. Still, we acknowledge this as a limitation and encourage future studies to incorporate within-sample reliability testing to further strengthen the confidence in outcome precision.

Another important limitation is the relatively small sample size, which may limit the generalizability of the findings. Although the observed trends were consistent and aligned with previous research, the results should be interpreted with caution and considered preliminary until replicated in larger and more diverse populations.

Finally, the participants in this study were young, resistance-trained individuals, which limits the generalizability of our results to older adults or untrained populations. Future research could explore these findings in other compound exercises and analyze the impact of accumulated fatigue on propulsive velocity across sexes.

## 7. Conclusions

In conclusion, the results of this study indicate that significant differences in strength and power relative to body mass and Fat-Free Mass (FFM) were found between males and females at loads ranging from 45% to 75% of the 1RM, reflecting underlying physiological and neuromuscular disparities. However, these differences disappear at 85% of the 1RM, likely due to maximal motor unit recruitment and similar neuromuscular strategies between sexes. These findings highlight the importance of designing sex-specific training programs to optimize performance based on both sex and load intensity.

## Figures and Tables

**Table 1:** Technical characteristics of the T-Force device.

Technology	Linear velocity transducer
Support APP on mobile	No
Software version	3.6
Indirect outcome calculation	Velocity; Time
Maximal Sampling frequency	1000 Hz
Mechanic’s parameters	Peak force, mean velocity, mean power, time to peak power, propulsive phase’s duration, estimated load (%1RM), 1RM prediction, number of repetitions, velocity loss (%), and velocity alerts. Automatically computed and presented numerically and graphically.
Screen	OLED Screen
Export to Excel	Yes
Bluetooth/WIFI connection	No
External power supply required	Yes
Installation and calibration time before the first execution	2.4 min
Time to obtain the measure after execution	Real time
Number of lost repetitions per each 100 cases	0.8
Price	2600 €

**Table 2:** Descriptive characteristics of the participants.

	Mean ± SD n Total	Mean ± SD Males	Mean ± SD Females
Age (*n* = 29)	21.9 ± 4.5	21.3 ± 4.1	22.6 ± 5.0
Height (cm)	171.9 ± 8.9	176.67.2	165.8 ± 7.1
Weight (kg)	67.8 ± 12.8	75.9 ± 10.1	57.9 ± 7.9
BMI (kgּ·m^−2^)	22.8 ± 2.7	24.3 ± 2.7	21.0 ± 1.3
Fat Mass (%)	16.1 ± 4.8	13.3 ± 2.7	19.5 ± 4.6
FFM (kg)	57.3 ± 11.8	66.1 ± 7.8	46.4 ± 4.4
1RM (kg)	73.1 ± 26.3	92.0 ± 19.7	49.7 ± 8.1

BMI: Body Mass Index, FFM: Fat-Free Mass, 1RM: One Repetition Maximum.

**Table 3:** Mean ± standard deviation values for mean propulsive velocity, strength and power in men and women in bench press.

Variable	Males	Females
45%1RM (m·s^−1^)	0.79 ± 0.06	0.73 ± 0.15
55%1RM (m·s^−1^)	0.66 ± 0.05	0.61 ± 0.12
65%1RM (m·s^−1^)	0.54 ± 0.04	0.47 ± 0.05
75%1RM (m·s^−1^)	0.46 ± 0.05	0.41 ± 0.04
85%1RM (m·s^−1^)	0.34 ± 0.04	0.32 ± 0.05
V_0_ (m·s^−1^)	1.36 ± 0.11	1.20 ± 0.27
F_0_ (N)	1.036.47 ± 185.38	568.18 ± 107.15
V_opt_ (m·s^−1^)	0.68 ± 0.06	0.60 ± 0.13
P_max_ (W)	349.73 ± 64.29	166.66 ± 26.76
%RM	57.18 ± 1.56	58.61 ± 3.42
Opt. Load (kg)	52.83 ± 9.44	28.96 ± 5.41

1RM: One Repetition Maximum, V0: Initial velocity, F0: Initial force, Vopt: Optimal velocity, Pmax: Maximal power, Opt. Load: Optimal load.

**Table 4:** ANCOVA results among sexes for the five different loads by two different adjustments.

Variable	Adjusted by Body Mass				Adjusted by FFM			
	F	*p*	η^2^	95%CI	F	*p*	η^2^	95%CI
45%1RM	3.309	0.080	0.113	0.713–0.796	5.906	0.022	0.185	0.716–0.797
55%1RM	3.529	0.072	0.120	0.596–0.666	5.190	0.031	0.166	0.597–0.667
65%1RM	5.971	0.022	0.187	0.491–0.525	16.118	0.001	0.383	0.493–0.526
75%1RM	4.394	0.046	0.145	0.417–0.452	7.449	0.011	0.223	0.417–0.453
85%1RM	1.390	0.249	0.051	0.309–0.345	1.664	0.208	0.060	0.308–0.346

1RM: One Repetition Maximum, η^2^ = Partial eta squared, ANCOVA = Analysis of Covariance. FFM: Fat-Free Mass.

## Data Availability

All data is presented in this manuscript.
